# The two faces of titanium dioxide nanoparticles bio-camouflage in 3D bone spheroids

**DOI:** 10.1038/s41598-019-45797-6

**Published:** 2019-06-27

**Authors:** W. Souza, S. G. Piperni, P. Laviola, A. L. Rossi, Maria Isabel D. Rossi, Bráulio S. Archanjo, P. E. Leite, M. H. Fernandes, L. A. Rocha, J. M. Granjeiro, A. R. Ribeiro

**Affiliations:** 1 Directory of Life Sciences Applied Metrology, National Institute of Metrology Quality and Technology, Rio de Janeiro, Brazil; 2 Postgraduate Program in Biotechnology, National Institute of Metrology Quality and Technology, Rio de Janeiro, Brazil; 3 Brazilian Branch of Institute of Biomaterials, Tribocorrosion and Nanomedicine (IBTN), Bauru, Brazil; 4 Brazilian Center for Research in Physics, Rio de Janeiro, Brazil; 5 Postgraduate Program in Translational Biomedicine, University Grande Rio, Duque de Caxias, Brazil; 6 Institute of Biomedical Sciences, Clementino Fraga Filho University Hospital, Federal University of Rio de Janeiro, Rio de Janeiro, Brazil; 7 Materials Metrology Division, National Institute of Metrology, Quality, and Technology, Rio de Janeiro, Brazil; 8 Institute of Biophysics Carlos Chagas Filho, Federal University of Rio de Janeiro, Rio de Janeiro, Brazil; 9 Faculty of Dental Medicine, University of Porto, Porto, Portugal; 10 Physics Department, Universidade Estadual Paulista, São Paulo, Brazil; 11 Dental School, Fluminense Federal University, Niterói, Brazil; 12 LAQV/REQUIMTE, University of Porto, Porto, Portugal

**Keywords:** Implants, Cell-particle interactions

## Abstract

Titanium (Ti) and its alloys are widely used in dental implants and hip-prostheses due to their excellent biocompatibility. Growing evidence support that surface degradation due to corrosion and wear processes, contribute to implant failure, since the release of metallic ions and wear particles generate local tissue reactions (peri-implant inflammatory reactions). The generated ions and wear debris (particles at the micron and nanoscale) stay, in a first moment, at the interface implant-bone. However, depending on their size, they can enter blood circulation possibly contributing to systemic reactions and toxicities. Most of the nanotoxicological studies with titanium dioxide nanoparticles (TiO_2_ NPs) use conventional two-dimensional cell culture monolayers to explore macrophage and monocyte activation, where limited information regarding bone cells is available. Recently three-dimensional models have been gaining prominence since they present a greater anatomical and physiological relevance. Taking this into consideration, in this work we developed a human osteoblast-like spheroid model, which closely mimics bone cell-cell interactions, providing a more realistic scenario for nanotoxicological studies. The treatment of spheroids with different concentrations of TiO_2_ NPs during 72 h did not change their viability significantly. Though, higher concentrations of TiO_2_ NPs influenced osteoblast cell cycle without interfering in their ability to differentiate and mineralize. For higher concentration of TiO_2_ NPs, collagen deposition and pro-inflammatory cytokine, chemokine and growth factor secretion (involved in osteolysis and bone homeostasis) increased. These results raise the possible use of this model in nanotoxicological studies of osseointegrated devices and demonstrate a possible therapeutic potential of this TiO_2_ NPs to prevent or reverse bone resorption.

## Introduction

In the growing scenario of nanotechnology, titanium and its alloys are the metallic materials widely used in dental and orthopaedic implant applications due to their excellent biocompatibility^1,2^. However, a significant number of dental implants (5–11%) and hip prostheses (65%) fail with quite harmful consequences for patients. The failure of the implant is multifactorial, in many cases related to infections, adverse biological responses induced by the by-products and patient’s health^1–5^.

Excessive physiological loads together with corrosion processes during/after implantation lead to surface degradation contributing also to implant failure^1,2^. In fact, when implanted, titanium may undergo the combined action of corrosion and wear mechanisms (tribocorrosion), which results in the release of metal ions as well as wear debris (micron and nanoparticles (NPs)) that alter local cell and tissue homeostasis^3–5^. Tissue homeostasis is addressed by an intensive cell cross-talk, where tissue repair and remodeling involve well-organized cells actions and appropriate chemokines, cytokines, growth factors as well as secretion of ECM proteins^6–9^. Current theories forecast a simultaneous activation of inflammatory mediators with protective and regenerative mechanisms in order to restore the original bone tissue architecture and normal metabolism. Consequently, depending on their size, debris can be internalized, generating several cell adverse reactions such as cytotoxicity, oxidative stress as well as DNA damage^10,11^. Occasionally, this debris can trigger an autoimmune response^12,13^. The inflammatory response generated by wear particles is mediated by the resident or infiltrating osteoblasts, osteoclasts, macrophages, and dendritic cells in the peri-implant tissue. It is very well documented that wear particles stimulate periprosthetic cells to secrete pro-inflammatory/pre-osteoclastic cytokines and chemokines that orchestrate and inhibit osteoblast activity with intensification and survival of osteoclast activity that ultimately leads to bone resorption^9,12,13^. The current literature supports that the released cytokines can modulate osteoclastogenesis, favouring periprosthetic bone resorption (osteolysis) that lead to pain, reduce patient motility, and ultimate in implant loss, with the need of a revision surgery^4,12^.

Traditional *in vitro* nanotoxicology studies rely on 2D cell culture monolayers as well as *in vivo* studies that enable the study of the direct interaction of nanoparticles with cells, while animal studies offer information on biodistribution, efficacy and toxicity^4,6,10^. However high controversy in the published results is observed due to the use of different cell culture models, lack of standardization of parameters such as nanoparticle size, agglomeration state, high exposure doses and times as well as interference of nanoparticles with the biological assays^14–17^. None of the 2D and 3D models explores the interactions with tissues in a controlled manner. Cell culture approaches in 3D have been established and are gaining importance since they induce cellular polarity, promote cell-cell interactions, and increase cell-cell and cell-extracellular matrix (ECM) adhesion/signaling, leading to the expression of specific genes/proteins and the consequent formation of a structure with *in vivo* tissue-like morphology^18–21^. The *in vitro* studies of nanoparticle effects in a 3D cell culture model seems to be more appropriate compared to 2D monolayer systems since toxicity results can be more strongly influenced by cell microenvironment^22–25^. The fundamental mechanisms of the physical interactions of NPs with cells, the ECM and concentration gradients are explored in 3D culture models. Over the last 60 years, spheroids have become an important part in understanding cell-cell and cell-matrix interactions and have been used as a valuable tool for studies of drug discovery and toxicological screening of drugs, where sometimes nanoparticles (NPs) are used as carriers, minimizing the number of whole-animal studies^18,26^.

The role of nanoparticles released by the degradation of an implant system in macrophages/monocytes (2D cell models) is already very well described, however, their direct effect on bone cells in a 3D microenvironment and its contribution to osteolysis is still a mystery. We believe that osteoblast spheroids can mimic the complex cell-cell interaction, and cell-ECM interaction making it an attractive new and promising approach to study the complex interactions of nanometric debris with the biological system^27,28^.

Taking this into consideration, in this work, we investigated the effect of TiO_2_ NPs interaction in bone homeostasis using a human osteoblast-like spheroid culture model. Cell viability, differentiation, mineralization and cytokine secretion upon nanoparticle exposure were evaluated and the obtained results open venues for the use of this system for regenerative purposes. Although clinicians mainly recognize nanoparticles as possible toxic agents, scientists can start to use their biological effects to improve the interaction of medical devices with surrounding tissues and cells.

## Results

### Anatase nanoparticles form stable aggregates in cell culture medium

**Figure 1:**
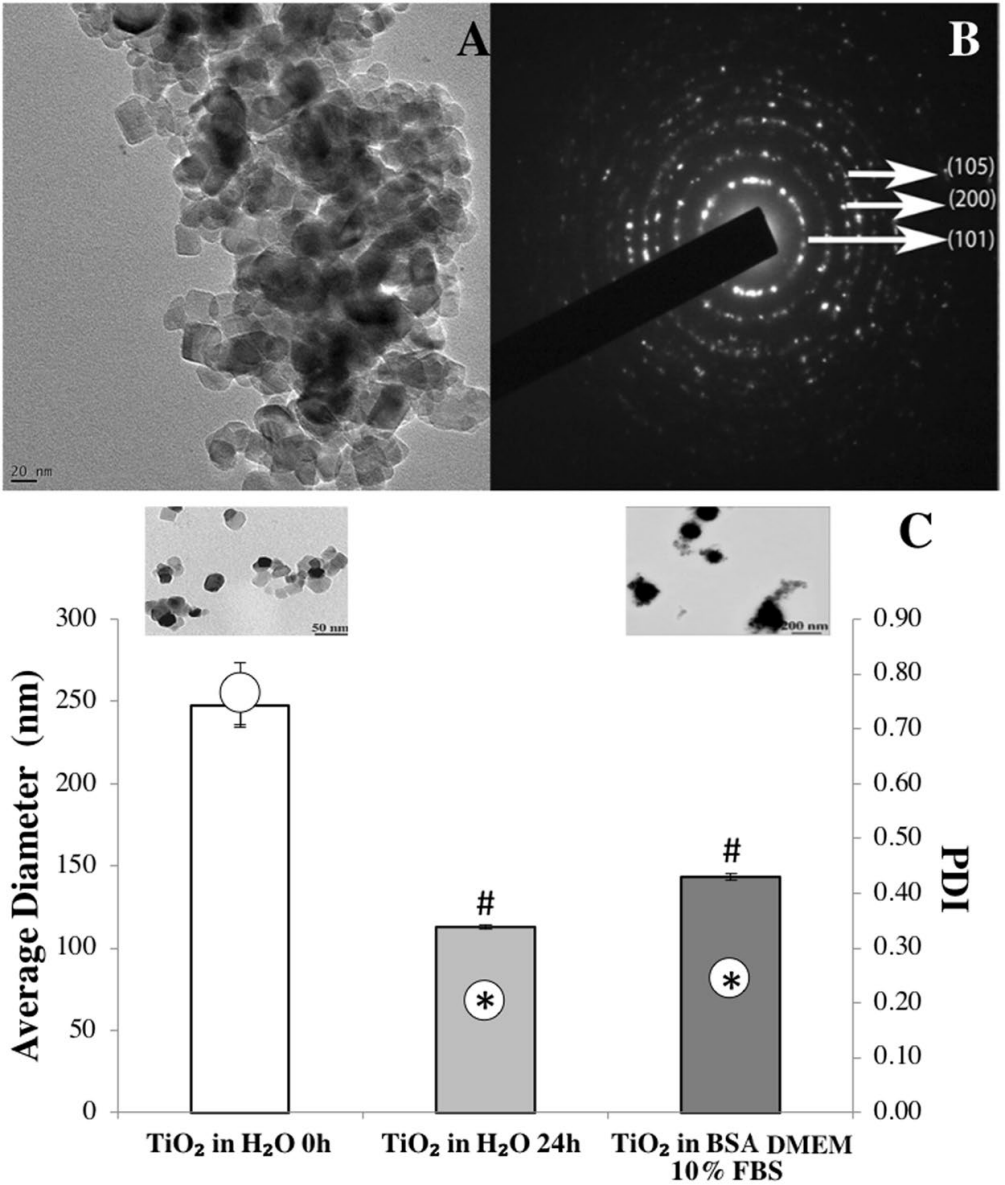
Physicochemical characterization of TiO_2_ nanoparticles: (**A**) Transmission electron micrograph showing the primary size of TiO_2_ NPs and their agglomeration due to their high reactivity. (**B**) Crystalline phase identification of anatase by electron diffraction obtained in TEM. (**C**) Average diameter and polydispersity index (PDI) of TiO_2_ NPs after dispersion in water (0 h, control), 24 h after stabilization and incubated with medium culture (TiO_2_ NPs in BSA DMEM 10% FBS). TEM micrographs of TiO_2_ NPs in distilled water and in medium culture are shown demonstrating an alteration in NPs morphology. Data represent mean ± standard deviation (SD), n = 30 in each condition. ^#^P < 0.05 vs control and *P < 0.05 vs control.

The primary size of the TiO_2_ NPs used in this work is 25 nm. However, most particles appear as agglomerates as observed in the transmission electron microscopy (TEM) micrographs presented in Fig. 1A. The crystalline structure, analyzed by electron diffraction, evidenced the presence of reflections corresponding to anatase (Fig. 1B).

A dispersion protocol using sonication was optimized and implemented in order to reduce the agglomerates size^13^. After dispersion in water and 24 h of stabilization, TiO_2_ NPs suspension was homogeneous (low polydispersive index (PDI)) presenting an average diameter of 113 ± 1 nm. The size obtained by dynamic light scattering (DLS) was in accordance with that observed by TEM (see insert in Fig. 1C). A significant increase in the mean diameter of TiO_2_ NPs, was observed when nanoparticles were added to the cell culture medium (data not shown). The addition of bovine serum albumin (BSA) as a stabilizing agent into the culture medium, significantly reduced the agglomeration, where TiO_2_ NPs present an average size of 143 ± 5 nm (Fig. 1C) prior to biological tests.

### TiO_2_ NPs exposure alters cell cycle distribution in the 3D environment upon nanoparticles uptake

**Figure 2:**
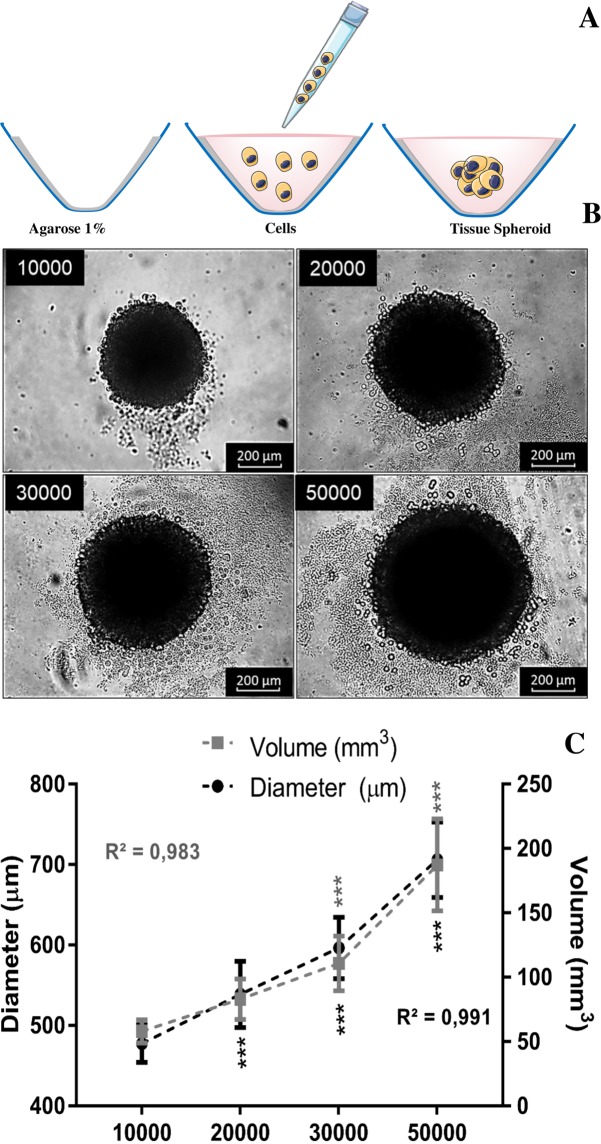
Establishment and characterization of human osteoblast spheroids: (**A**) Schematic representation of spheroids formation after aggregation and compaction of cell suspension cultured in nonadherent conditions (**B**) phase contrast images obtained by optical microscopy demonstrating the growth of spheroids produced with different numbers of cells (10000, 20000, 30000 and 50000 cells). (**C**) Spheroid diameter and volume is plotted against the different number of plated cells from three independent experiments (n = 10). Data are shown as mean ± SD. Spheroid diameter and volume was calculated based on phase contrast image analysis by area determination using image J software. A p-value < 0.05 is marked as statistically significant (***).

To develop and optimize the three-dimensional model, we use different cell numbers (10000, 20000, 30000 and 50000 cells) of a human osteoblast-like cell line (SAOS-2) that was seeded in round bottom 96 well plates previously covered with a thin layer of agarose to prevent cell adhesion to the plate. After three days of culture, spheroids were formed (see schematic representation in Fig. 2A). The round shape morphology of the spheroids was maintained independently of the cell number (Fig. 2B). As expected, the diameter and volume of spheroids increased linearly with the number of plated cells, as it is shown in Fig. 2C.

**Figure 3:**
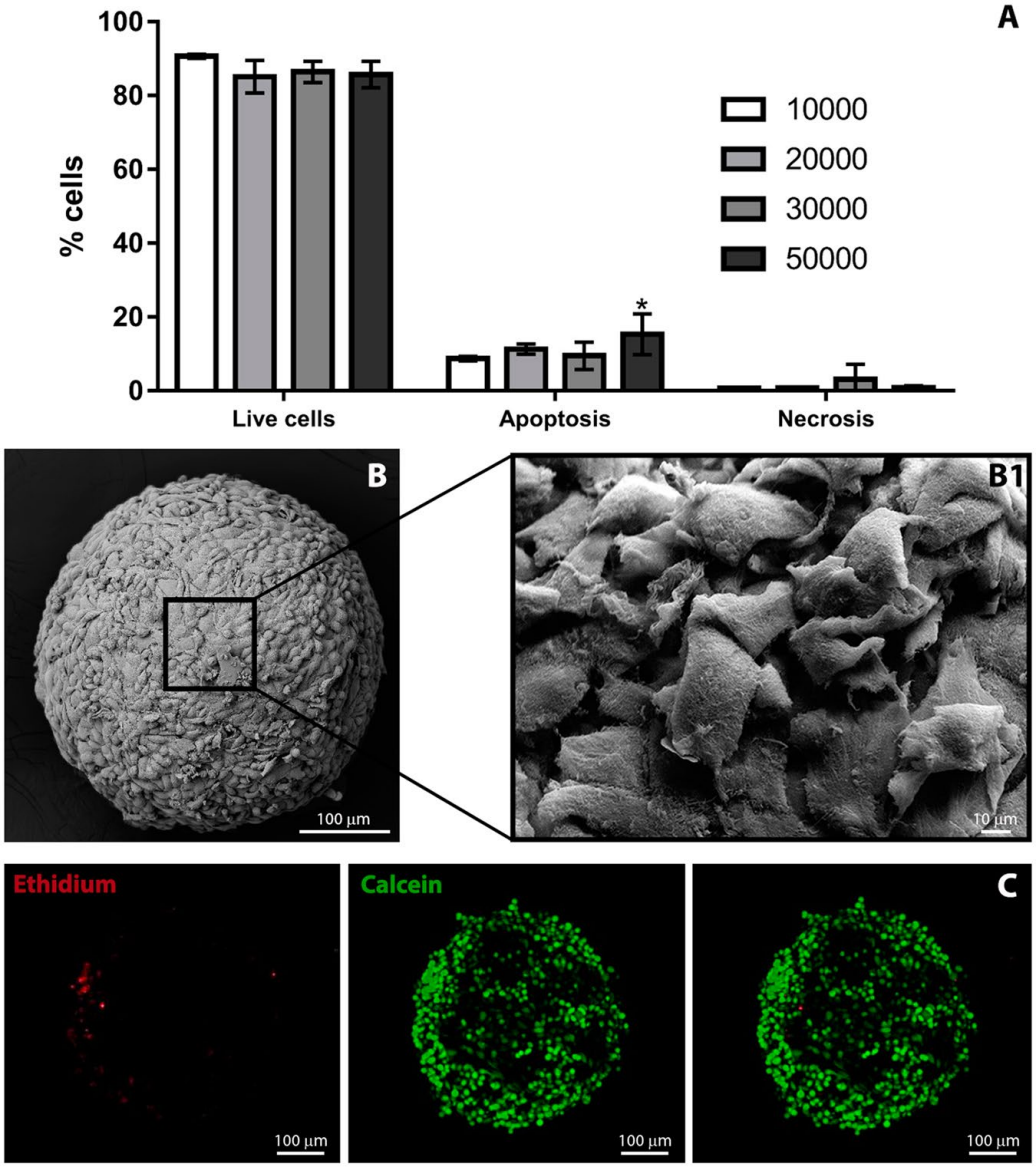
Spheroids characterization: (**A**) Cell viability of spheroids produced with different cell numbers measured by Dead Cell Apoptosis Kit in flow cytometry. Bars are shown as mean ± SD, calculated from a group of 12 spheroids for each condition. *P < 0.05 vs control. (**B**) Morphology of the spheroids produced with 10000 cells obtained by scanning electron microscopy where high magnification images demonstrate cell-cell interactions (B1). (**C**) Confocal microscopy of a live spheroid stained in red with ethidium-positive (dead cells) and in green with calcein-positive (live cells), merged image demonstrating the presence of live and dead cells. The fluorescence intensity profiles for both channels show the different distribution of live and dead cells in the spheroid structure.

Although no significant differences were observed in the percentage of viable cells, for all the conditions (Fig. 3A), spheroids formed with 10000 cells showed viability close to that observed in monolayers and were thus selected for further investigations. The spherical shape of spheroids formed with 10000 osteoblasts was confirmed by scanning electron microscopy that revealed closely cell-cell interactions between osteoblasts located at the external layer (Fig. 3B). The majority of the cells in the inner layer of spheroids were viable (Fig. 3C).

**Figure 4:**
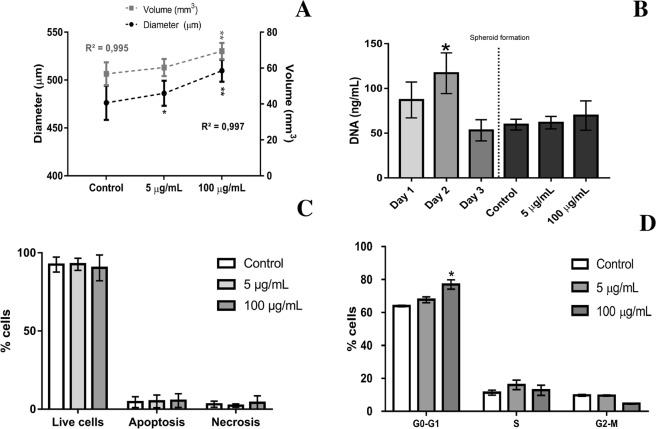
Spheroid size, proliferation, viability and cell cycle analysis after nanoparticles exposure (5 and 100 µg/ml of TiO_2_ NPs for 72 hours): (**A**) Spheroid diameter and volume of 10,000 cells spheroids upon NPs exposure was calculated based on phase contrast image analysis by area determination using image J software. Data are shown as mean ± SD of three independent experiments (n = 10). (**B**) spheroid proliferation during formation and NPs exposure. (**C**) Annexin V/PI assay captures the different cell death profiles in the spheroids exposed to different concentrations of TiO_2_ NPs. (**D**) cell cycle analysis showing percentages of Go-G1 phase, S and G2/M phase of spheroids exposed to NPs. Bar represents the mean ± SD performed with 4 spheroids in each condition and the analyses were performed in triplicate. * and **P < 0.05 vs control.

Next, we addressed how TiO_2_ NPs influenced osteoblasts-like functions. Exposure of osteoblasts spheroids to TiO_2_ NPs led to an increase in the diameter and volume after 72 h (Fig. 4A), however, no significant differences were observed on spheroid proliferation (Fig. 4B) suggesting nanoparticles internalization. No significant differences were observed in cell viability inside the spheroids after exposition to TiO_2_ NPs (Fig. 4B,C). Nonetheless, cell cycle analysis revealed a significant increase in the percentage of cells in G1 phase as the concentration of TiO_2_ NPs increased (Fig. 4D). No significant differences in the percentage of cells in the S and G2-M phase were observed after TiO_2_ NPs exposition when compared to the control (Fig. 4D).

### Uptake of nanoparticles in 3D spheroid influences ECM secretion/deposition, cytokine and secretion of growth factors

**Figure 5:**
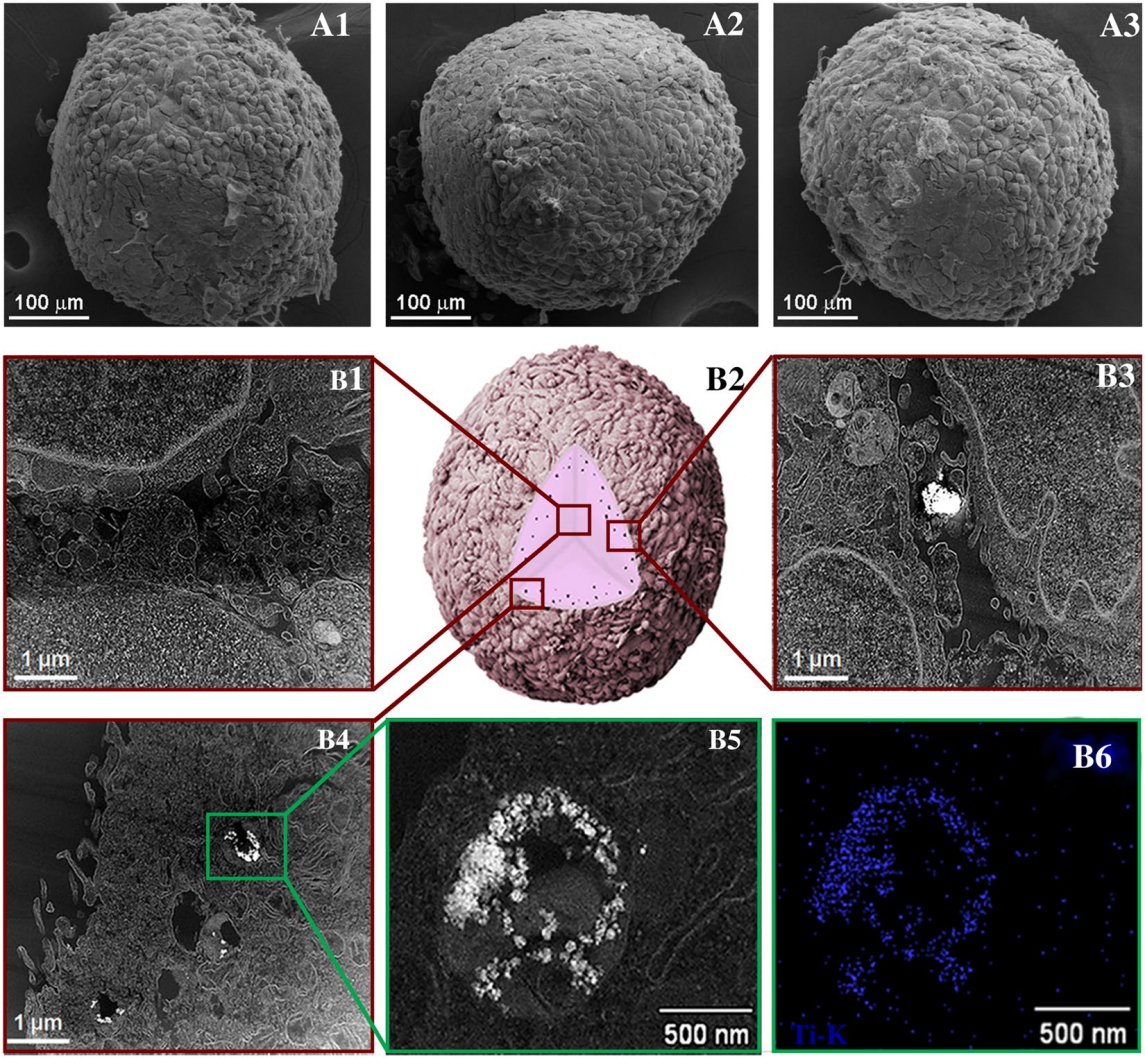
Spheroid organization and TiO_2_ NPs internalization: (**A1**) SEM micrograph of spheroids in the control condition (without NPs); (**A2**) with 5 and (**A3**) 100 µg/ml TiO_2_ NPs exposure for 72 hours. (**B1–B6**) Internalization and distribution of TiO_2_ NPs in spheroids exposed to 100 µg/ml NPs; (**B1**) Scanning TEM (STEM) micrograph of the interior of the spheroids. (**B2**) Schematic illustration of the penetration behavior of TiO_2_ NPs, (**B3**) STEM micrograph of the outer layers of the spheroid showing TiO_2_ NPs in the space between cells, (**B4**) and in membrane-vesicle, (**B5**) high magnification of the membrane vesicle, (**B6**) STEM/EDS map of Ti-K X-ray line confirming the presence of TiO_2_ NPs. Images are representative of four independent analysis.

We then examined the internalization of TiO_2_ NPs with osteoblasts and their effect on differentiation and mineralization. The morphology of the spheroids after TiO_2_ NPs exposure was evaluated by SEM and TEM, as shown in Fig. 5. Spheroids maintained their spherical shape after TiO_2_ NPs exposure. There was no evidence of the presence of TiO_2_ NPs in the centre of the spheroid (Fig. 5B1). For all the concentrations tested, TiO_2_ NPs were observed preferentially located in the outer layers of the spheroid in spaces between cells (Fig. 5B3) as well as inside of the cells in well-defined membrane vesicles (B4, B5). The presence of TiO_2_ NPs was confirmed by X-ray energy dispersive spectroscopy (EDS) chemical analysis (Fig. 5B6).

**Figure 6:**
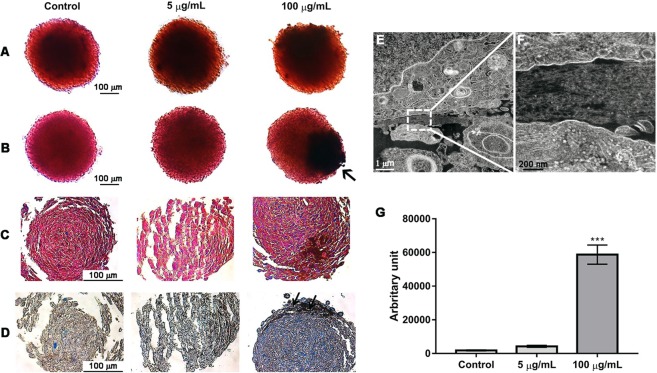
Spheroid differentiation, mineralization and matrix organization after TiO_2_ NPs exposure (5 and 100 µg/ml of TiO_2_ NPs during 72 hours): cross sections of paraffin-embedded spheroids stained with: (**A**) alkaline phosphatase for differentiation, (**B**) Alizarin Red S to visualize calcium deposition, (**C**) and Masson’s trichrome and (**D**) aniline blue staining for collagen. Images shown are representative of three independent experiments with triplicates. The arrows indicate the presence of TiO_2_ NPs. (**E**,**F**) TEM image of an ultrathin section of an osteoblast spheroid where inset delineated by a dotted line in E is magnified in F and demonstrate the presence of collagen fibrils. (**G**) Quantification of collagen deposition was performed by Blue pixel analysis and quantification in aniline blue sections using Image J software. Data show mean ± SD of three independent experiments in triplicates for each condition. *P < 0.05 vs control.

The influence of TiO_2_ NPs on spheroid differentiation and mineralization was evaluated by alkaline phosphatase and alizarin red assays, respectively (Fig. 6A,B). Results revealed that exposure to TiO_2_ NPs did not influence differentiation and mineralization of spheroids (see (Fig. 1 in complementary information that demonstrates the quantification of ALP activity). A darker colouration (indicated by an arrow in Fig. 6B) was observed, which may be the result of TiO_2_ NPs accumulation and diffusion in the spheroid. Masson’s Trichrome and aniline blue staining for collagen (Fig. 6C,D) revealed an increase in collagen deposition in spheroids exposed to 100 µg/mL TiO_2_ NPs (Fig. 6G). This result was confirmed by TEM, where a dense matrix between cells was observed (Fig. 6E,F).

**Figure 7:**
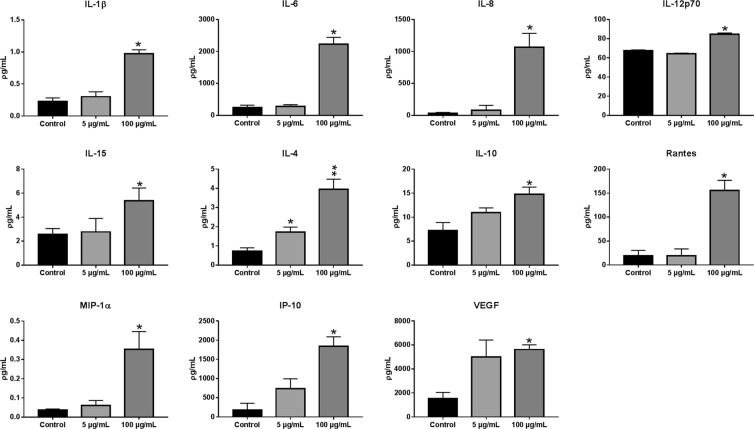
Secreted inflammatory mediators by osteoblasts upon TiO_2_ NPs exposure (5 and 100 µg/ml of TiO_2_ NPs during 72 hours): After TiO_2_ NPs treatment, the supernatant was collected and the secreted inflammatory mediator’s analyzed. Data show mean ± SD of three independent experiments. *P < 0.05 vs control.

The inflammatory response of osteoblasts to TiO_2_ NPs was evaluated. Significant differences in the amount of the cytokines (IL-1B, IL-6, IL-8, IL-12, IL-15, IL-4, IL-10), chemokines (Rantes, MIP-1a, IP-10) and growth factors (VEGF) were observed in the supernatant of the spheroids incubated with 100 μg/mL TiO_2_ NPs in comparison with the control (without TiO_2_ NPs) (Fig. 7). Most of the cytokines and growth factors have a dual effect and are involved in osteolysis and bone homeostasis^4,12^.

## Discussion

Osseointegrated implants can undergo the combined action of corrosion due to the presence of corrosive biological fluids and wear mechanisms (tribocorrosion) through small amplitude oscillatory movements as a result of physiological loads that may take place at implant/bone interface. This becomes an issue of high clinical relevance once Ti-based materials display poor wear resistance^1–3^. In the tissues surrounding dental implants and hip prostheses, metal-like particles were histologically observed^14,29^. As a consequence of this metal ions and wear particles, there is the peri-implant inflammatory reactions with consequent aseptic osteolysis^3,5,10,14^. The release of TiO_2_ micron particles may also compromise bone-forming cell functions and bone remodelling, however, the information regarding the effect of nanosized TiO_2_ particles in 3D bone cells spheroids is absent^13,15,30–35^.

Three-dimensional (3D) models have been used in the area of cancer and bioengineering, however, for nanoparticle safety assessment, they are still unexplored^21–25,36–39^. Although 3D cell culture systems do not replace *in vivo* studies, they can bridge the gap between traditional 2D *in vitro* studies and *in vivo* models^18,27,37,40–43^. The main limitations of 2D models for NPs safety assessment are: absence of tissue architecture, cell polarization and spatial organization of surface receptors, cell communication (most of *in vitro* studies are single cell type), differences in cell stages (3D cultures usually are a mixture of cells at different stages) and sedimentation effect of NPs on top of the cells that limit their accessibility and diffusion^20,44,45^. Spheroids simulate the complex cell organization established *in vivo* becoming an attractive and promising approach to study the complex interactions of TiO_2_ NPs in a 3D environment. The model developed in this work has the advantage of presenting a tissue-like organization where the close contact of spheroids cell surface and NPs is driven by occasional movement, avoiding sedimentation and mass effect that is known to give rise to underestimated or exaggerated toxicity estimations^23^. Besides that, it can be used as a model system for understanding tissue-level healing processes, since the osteoblastic-like spheroid release cytokines, chemokines and growth factors, suggesting a capacity of the model for physiological repair, which is closer to real tissue biology.

Our study is the first which study the effect of TiO_2_ NPs in human osteoblast-like spheroids. The spheroids chosen for toxicological evaluation were the smallest (approximately 485 μm of diameter) since they presented the highest viability (obtained by cytometry and confocal microscopy with live/dead tag) and lower degree of apoptosis and necrosis possibly due to their size. From literature, it is observed the formation of necrotic cores in larger spheroids due to restricted oxygen, nutrients and metabolites diffusion that also compromise cell survival^18,26,37,40,46,47^. Regarding spheroid morphology it was possible to observe its round shape, but most important was the establishment of close cell-cell, cell-ECM interactions and collagen secretion (confirmed by histology, SEM and TEM analysis). The round spheroid shape normally indicates strong cell–cell adhesion that usually expresses tight cell junction^20^. Proximal cell-cell interactions in the sphere-shaped model also endorse the recovery of native tissue morphology and function. In natural tissues, the ECM with its biochemical and biophysical signals regulate homeostasis, in spheroids is an important key factor that maintains their structural integrity, and the establishment of a matrix-dependent communication network^37,40–42,48^. The reduction of cells proliferation during spheroid formation is possibly caused by spheroid maturation processes where the secretion of their own ECM and cells re-arrangement (tightened cell-cell and cell-ECM interactions) are characteristic features^49–51^.

No significant morphological differences were observed in the spheroids upon TiO_2_ NPs exposure besides an increase in diameter and volume, that was not related to spheroid proliferation but rather possibly related with TiO_2_ NPs internalization and collagen secretion. We visualize TiO_2_ NPs preferentially internalized by cells in the outer layers of the spheroids in specific membrane vesicles, although some TiO_2_ NPs were observed in spheroid’s interstitial spaces. Perhaps the extracellular matrix secretion in the 3D model is contributing to the structural integrity of the spheroid, but also to the regulation of TiO_2_ NPs diffusion within the interstitium (ECM work as a natural barrier possibly limiting NP diffusion into the tissue)^3,38,52^. The mechanism of TiO_2_ NPs internalization in human bone cells was already reported and it is related with the calcium and phosphorous rich bio-camouflage formed around NPs that facilitates NPs internalization (works as a Trojan horse mechanism of internalization)^13^.

Although there is some evidence about a size–toxicity relationship for some nanoparticles, for TiO_2_ NPs this evidence remains inconclusive^53,54^. In this work, no significant differences in viability, apoptosis and necrosis were observed after the short exposure time (72 h), despite the fact that TiO_2_ NPs were internalized by cells. This result is in agreement with 2D literature that shows that TiO_2_ NPs exposure to primary human osteoblasts^13^ and SAOS-2 didn’t affect their viability^35^. Normally 3D models used for nanoparticles safety assessment normally reports inferior nanotoxicity when comparing with 2D models, however, there is no information is available in the literature regarding the effect of TiO_2_ NPs in osteoblasts-like spheroids. A study using the same nanoparticles reveals a slight decrease in human alveolar epithelial cells spheroids viability where TiO_2_ NPs affect cell-cell interaction during spheroid formation^36^. More interesting was the observed changes in the cell cycle. The cell cycle is divided into different phases: cells divide in the M phase, while protein synthesis and DNA duplication mainly happen in G1 phase and S phase, respectively. The progress of the cell cycle can be significantly changed by exogenous substances such as NPs^54^. Cells with damaged DNA will be arrested in any of the phases referred before depending on the intensity of damage. In this study, it was possible to observe a significant increase in the percentage of cells in G1 phase for higher TiO_2_ NPs concentration exposure. Increase in cell size and centrosome duplication, synthesis of RNA and proteins for DNA formation are initiated in G1 phase. Studying the influence of nanoparticles on the G1-phase of the cell cycle is essential since it is a period in which cells decide whether to start proliferation or stay quiescent. The arrest of TiO_2_ NPs exposed cells in G1 phase imply a possible inhibition of DNA synthesis with a consequent reduction on cell proliferation^55^. It is proposed that TiO_2_ NPs might potentially induce cell cycle modifications due to their ability to produce free radicals that can damage DNA by oxidation, nitration, methylation or deamination reactions^2,56–58^. Long-term cultures, as well as clonogenic assays, could increase the understanding of the reported results.

In this work, we have demonstrated that TiO_2_ NPs didn’t affect differentiation and mineralization, however, it stimulates collagen deposition in spheroids. In fact, this result is interesting, since collagen is the most abundant ECM protein in bone. 3D cell models are well known for their ability to dynamically produce their own ECM^43,59^. Similar to our findings, iron oxide nanoparticles stimulate the synthesis of collagen in rat aortic smooth muscle spheroids^60,61^ and silver NPs regulate collagen deposition and alignment in the wound healing model^62^.

The mechanism involved in this increased collagen deposition upon exposure to TiO_2_ NPs is possibly related with the bio-camouflage reported before as well as the secretion of vascular endothelial growth factor (VEGF) induced by TiO_2_ NPs internalization. The calcium and phosphorous adsorbed ions present in NPs bio-camouflage, are fundamental for collagen secretion, bone mineralization and bone metabolism^63–67^. VEGF is a potent mediator of angiogenesis, also critical for bone development and regeneration^64,68–72^, however, its overexpression can contribute to bone resorption due to osteoclast recruitment^73^. In 3D models, spheroids (>100 µm) exhibit a more pronounced upregulation of VEGF secretion when compared to smaller ones^59,60,71^ and there is a correlation in the decrease of VEGF secretion together with the reduction of collagen deposition^72^. Interestingly, *in vitro* studies using 2D osteoblast cells demonstrate that phagocytose of TiO_2_ particles induce pro-inflammatory cytokine secretion with consequent suppression of collagen synthesis. However, this effect can be compensated and/or reversed by the release of growth factors such as insulin-like growth factor and transforming growth factor- ß1^6^. We believe that the 3D model used in this work presents an interesting microenvironment, whereupon TiO_2_ NPs internalization osteoblasts functions are altered secreting high concentrations of VEGF that can induce collagen secretion and possibly reverse the effect of pro-inflammatory cytokines. Molecular studies should be carried out to clarify this phenomenon.

The higher levels of cytokine expression for the elevated concentrations of NPs tested supports the direct involvement of osteoblastic cells in inflammatory processes in response to wear debris. In aseptic loosening phagocytosis of wear debris, activated macrophages secrete mediators of bone resorption such as interleukin 1ß (IL-1ß, a key molecule in tissue remodelling), IL-6, IL-8 and tumour necrosis factor-alpha (TNF-alpha). This results in monocyte recruitment, suppression of osteoblastic differentiation and induction of osteoclastogenesis^12,13,52,74^. In this work, the highest levels of cytokines released by spheroids of osteoblasts upon exposure to TiO_2_ NPs and present in the supernatant were observed for IP-10, IL-6, and IL-8, data that is in agreement with the literature^58–75^. IL-6, TGF-ß and IL-10 (also detected on the supernatant) are considered as anti-inflammatory factors associated with bone homeostasis and, interestingly, IL-6 has a dual effect working as pro-inflammatory mediator associated with bone resorption^8^. Cytokines and chemokines such as IL-8, MIP-1alpha and Rantes (also detected for higher concentrations of TiO_2_ NPs) were observed in periprosthetic tissues, and they are known to amplify the inflammatory response providing a mechanism for resolution and repair^7^. IL-10 promotes osteoblastic differentiation, inhibiting collagenase synthesis and osteoclasts formation. It is considered as an anti-inflammatory cytokine playing an important role in downregulating inflammation in aseptic loosening^64,75,76^. IL15 (also detected in the supernatant) is also a pro-inflammatory cytokine observed in our experiments that are involved in bone mineralization and osteoblast function, playing an important role in determining osteoblast phosphate homeostasis and mineralization capacity^77^.

Summing up, our present data suggest that wear NPs released from the interface between the implant and bone may have a significant effect on osteoblasts. In the concentrations tested TiO_2_ NPs didn’t disrupt osteoblast viability. Interestingly, the bio-complex formed around NPs stimulate its internalization in the 3D microenvironment that induces the secretion of pro-inflammatory cytokines, chemokines and growth factors that contribute to the increase in collagen secretion/deposition. The cytokines and chemokines secreted by osteoblasts upon stimulation with TiO_2_ NPs are involved in the innate and adaptive immune responses, angiogenesis, wound healing, tissue repair but also in chronic inflammatory, playing an important role in bone resorption. Until now, it is unclear if the TiO_2_ NPs are stimulating an inflammatory or repair response since all the data suggest that the growth factors, cytokines and chemokines secreted upon exposure to high concentration of TiO_2_ NPs can have a dual effect. They are involved in both bone resorption and regeneration under specific circumstances. Although TiO_2_ NPs stimulate the secretion of chemokine and cytokines also detected in periprosthetic tissues, the potent growth factor secretion is possibly stimulating the collagen deposition that can perhaps protect against aseptic loosening in the earlier release of NPs from implants. Osteoblasts-secreted cytokines and chemokines may amplify and sustain osteoclastic bone resorption at the prosthetic interface. However, some of the detected cytokines have a dual effect. That is, they can either promote osteolysis or maintain bone homeostasis. We believe that the secretion of growth factors such as VEGF upon TiO_2_ NPs uptake by osteoblasts in the 3D model is increasing collagen deposition and so contributing to bone repair.

This work addresses the two “faces” of TiO_2_ NPs since we detect biological signals, both involved in bone resorption and bone homeostasis and regeneration^7^. We still need to clarify the mechanisms and explore the results as possible innovative regenerative strategies that use metal-based products for bone regeneration. Eventually, the local delivery of growth factors may potentially enhance initial osseointegration of implants and mitigate the adverse effects of wear debris, preventing periprosthetic bone loss.

## Conclusions

This article used 3D osteoblasts cells spheroids to evaluate cell viability upon exposure to TiO_2_ NPs, where spheroid morphology, pro-inflammatory cytokines and extracellular matrix proteins deposition were also studied. In the complex biological environment, TiO_2_ NPs form bio-complexes (ion and protein adsorption) resulting in a kind of Trojan horse that facilitates their internalization by 3D spheroid. The presence of the ionic bio-complex can activate several cellular functions such as mineralization, collagen deposition, and internalization of the TiO_2_ NPs in the spheroids of osteoblasts. Interestingly, in 3D spheroids, fully formed cell-cell and cell- ECM interactions in all three dimensions play a protective role and resulted in greater resistance to TiO_2_ NPs toxicity. The increase in the deposition of collagen, a protein present in the bone matrix, after exposition to the high concentration of TiO_2_ NPs, open the possibility of using this system in bone regenerative medicine. The development of this model may benefit the development of tissue engineering based on spheroids in future regenerative medicine.

## Materials and Methods

Titanium dioxide nanoparticles (TiO_2_ NPs anatase, Sigma-Aldrich) in the form of anatase, with a particle size <25 nm and surface area: 45–55 m^2^/g was used in this work. Osteoblasts were cultured in DMEM medium (Dulbecco’s Modified Eagle’s Medium, Lonza) supplemented with 10% (V/V) fetal bovine serum (FBS, Gibco). Bovine serum albumin (BSA, Sigma-Aldrich) dissolved in phosphate-buffered saline (PBS, Gibco) was used as a stabilizing agent during the anatase dispersion protocol.

### Physicochemical characterization of TiO_2_ NPs

For the evaluation of the size and morphology, TiO_2_ NPs were suspended in water (ultrapure water, Milli-Q) and subsequently analyzed by Transmission Electron Microscopy (TEM, JEOL 2100F) and by dynamic light scattering (DLS, Zeta-Sizer Nano ZS, Malvern Instruments GmbH). For all TEM analysis, one drop of the aqueous solution of TiO_2_ NPs was placed in a carbon-coated copper grid and air dried. The crystalline structure of nanoparticles was characterized by electron diffraction in TEM. DLS measurements were performed at 25 °C using 10 mm polystyrene disposable cuvettes and analysis were performed in triplicates.

### Titanium dioxide nanoparticles dispersion

A stock suspension of TiO_2_ NPs was prepared in ultrapure water (concentration of 2 mg/mL; pH 4). Following this procedure, an ultrasound (ultrasound, Q-Sonica) equipped with a 19 mm Ti tip was used to disperse TiO_2_ NPs. To reduce an excessive temperature rise, an ice bath was used to cool the suspension during the direct sonication process. The sonication was performed at 32 W of acoustic delivery power for 15 minutes in the pulse mode. After 24 h of stabilization, analysis of the particle size distribution was determined by DLS and TEM. Characterization of TiO_2_ NPs in the cell culture medium was performed by diluting the suspension of 2 mg/ml NPs in DMEM supplemented with 10% (v/v) FBS, pH 7.4.

### Spheroids development, viability studies and morphological analysis

#### Spheroid formation

The human osteoblast cell line SAOS-2 was supplied by the Cell Bank of Rio de Janeiro (Banco de células do Rio de Janeiro, BCRJ). They were packed in freezing ampoules and kept in liquid nitrogen. After thawing, the cells were expanded into 25 and/or 75 cm^2^ cell culture flasks (Corning) with DMEM medium supplemented with 10% FBS. The cells used in the experiments were between the 5° and 8° passage and were kept in a humidified incubator (5% CO_2_, 37 °C). The sterility test for bacteria, fungi and mycoplasma were performed in the beginning and end of all cell culture tests. For bacteria and fungi analysis, the cell culture supernatant was inoculated in Thioglycolate (TIO) (Acumedia) and tryptic soy broth (TSB) (Acumedia) during 14 days under aerobic conditions at temperatures: 22.5 °C ± 2.5 °C, TSB and 32.5 °C ± 2.5 °C respectively. The detection of mycoplasma contamination was performed in cell supernatants by bioluminescence, using the MycoAlert™ PLUS kit Mycoplasma Detection (MycoAlert®, Lonza). For spheroid formation, U-bottom plates (Corning) were coated with a thin layer of sterile ultrapure agarose (Sigma-Aldrich). Different SAOS-2 cell numbers (10000, 20000, 30000, and 50000 cells/well) were seeded in 200 μL DMEM medium supplemented with 10% FBS with 1% Penicillin/Streptomycin (PS - 10000 units/mL of penicillin and 10000 µg/mL of streptomycin) (PS, Gibco) in 96-well U-bottom plates that were and incubated at 5% CO_2_, 37 °C for 3 days, until the formation of the spheroids. The growth, shape and morphology of spheroids were monitored on the inverted optical microscope (Nikon Eclipse) and photographed using the photo program (ScopePhoto Leica software) using 10x magnification lens (LAS EZ, Leica). For each condition, 30 spheroids were photographed, and their diameter was measured with the help of a millimetre scale ruler using ImageJ program (version: 2015). In particular, for each spheroid, the volume was calculated considering spheroid diameter and applying the following mathematical formula (*V* = 4*π*r^3^/3).

#### Viability and proliferation studies

Flow cytometry was used to measure spheroids cell viability. Spheroids with different cell numbers (10000, 20000, 30000, and 50000 cells/well) were analyzed with Dead Cell Apoptosis Kit for Annexin V (Kit Life and Dead, Life Technologies). As a control, cultured cells (10000) were used in monolayer. The spheroids were washed 3 times with 0.01 M phosphate-buffered saline (PBS) then incubated with 0.125% Trypsin (kept in a humidified incubator 5% CO_2_, 37 °C) for 5 minutes. Trypsin was blocked with the addition of culture medium with 10% FBS and spheroids were mechanically dissociated. The cells were then centrifuged for 7 minutes at 500 g (4 °C), the pellet suspended in 100 μl annexin-binding buffer. The samples were incubated for 15 min (room temperature) in 3 μL annexin/Fluorescein (FITC) solution and 1 μL propidium iodide (following the instructions and volumes indicated by the manufacturer). For these analyses a pool with 4 spheroids of each condition was used and the analysis repeated in three independent experiments. All analyses were done in a flow cytometer (FACSAria III, BD Biosciences). The gating procedure applied to subsequent experimental data is presented in complementary information (Fig. 2). Another viability test was performed in spheroids of lower number of cells using Live/Dead viability/cytotoxicity Kit for mammalian cells (2 μM calcein AM and 4 μM EthD-1) (Kit Live/Dead®, Invitrogen Corporation). The spheroids were washed (3 consecutive times) with 0.01 M phosphate-buffered saline (PBS) and incubated (4 °C) with the Live/dead solution for 20 minutes. Calcein AM (494/517 nm green colour) marking live and ethidium homodimer-1 dead cells (528/617 nm red color). Spheroids were analysed and images acquired in a confocal laser scanning microscopy (Confocal laser scanning microscopy - DMI 6000, Leica).

#### Morphological studies

Spheroids (10000 cells) were placed in 0.01 M phosphate-buffered saline (PBS) and processed to scanning electron microscopy (SEM). Briefly, spheroids were fixed in modified Karnovsky’s solution (2% paraformaldehyde (PF, Sigma-Aldrich) 2.5% glutaraldehyde (glutaraldehyde, Sigma-Aldrich) in 0.1 M sodium cacodylate buffer (Buffer, Sigma-Aldrich), pH 7.2) for 1 hour at room temperature (RT) and washed with 0.1 M cacodylate buffer. The samples were post-fixed in a solution containing 1% osmium tetroxide (ReagentPlus^®^, Sigma-Aldrich) in cacodylate buffer (1:1) for 30 minutes (in the dark), washed with cacodylate buffer and dehydrated in a series of ethanol (Ethanol, VETEC) (30–100%). Spheroids were brought to the critical point (Critical point, Autosamdri-815a), mounted on aluminium stubs, and coated with gold (nm-thick layer of gold). The samples were analyzed SEM (JEOL JSM-6390LV with 5 kV).

### Nanotoxicological studies

#### Spheroid viability and proliferation

Spheroids (10000 cells) were exposed to TiO_2_ NPs (5 μg and 100 μg/mL) during 72 h and its viability was analyzed by flow cytometry following the protocol described before. Cell cycle analysis was performed by incubating spheroids with Hoechst 33342 (1 mg/ml, Molecular Probes, Eugene) at 37 °C during 15 min. All analyses were done in a flow cytometer (FACSAria III, BD Biosciences). The gating procedure applied in the subsequent experimental data is presented in complementary information (Fig. 3). For proliferation assays, cells were washed twice with PBS and then incubated in 0.1% Triton X-100 (TX-100, Sigma-Aldrich) for 10 min. DNA was quantified by the PicoGreen® dsDNA Quantitation Assay Kit (PicoGreen®, Invitrogen), according to the manufacturer’s instructions. Plate fluorescence was read on a microplate reader (Synergy HT, Bioek) using 480 nm excitation and 520 nm emission.

#### Spheroid morphology and internalization of TiO_2_ NPs

After TiO_2_ NPs exposure, the morphology of the spheroids was assessed by scanning electron microscopy (SEM) following the protocol described before. For transmission electron microscopy, spheroids were fixed in a modified Karnovsky’s for 2 hours at room temperature and washed with 0.1 M cacodylate buffer. The samples were post-fixed in a solution containing 1% osmium tetroxide and 0.8% potassium ferrocyanide (1:1) (Potassium ferrocyanide, Sigma-Aldrich) for 45 minutes (in the dark), washed with cacodylate buffer and contrasted with 1% uranyl acetate (diluted in water) (Uranyl acetate, Sigma-Aldrich) overnight. Samples were dehydrated in a series of ethanol (Ethanol, VETEC) (30–100%) and finally embedded in resin Epon 812 (EMS). Ultra-thin sections (70 nm) were cut in an ultramicrotome and examined using a transmission electron microscope (TEM, Tecnai Spirit G2, FEI). At least ten cells of each group (control, 5 μg/mL and 100 μg/mL) were analyzed. EDS in Scanning Transmission Electron Microscopy (STEM) mode was carried out using a Noran Energy EDX spectrometer that was applied to investigate regions containing nanoparticles inside of the cells. Specifically, a focused probe was raster scanned across the specimen, and at each probe position, the resultant X-ray emission spectrum was recorded.

#### Spheroid extracellular matrix organization and secretion/deposition

After treatment with TiO_2_ NPs, spheroids were stained with an alkaline phosphatase labelling kit (Leukocyte Alkaline Phosphatase, Sigma-Aldrich) based on the application of 500 μL of dioxin and naphthol saline in each well, leaving 30 minutes in the dark. After 30 min the reaction was quenched with distilled water. The resulting insoluble diffuse, red dye deposit indicates sites of alkaline phosphatase activity. For mineralization studies, cells were stained with 1% alizarin (red alizarin, Sigma-Aldrich) red solution. To analyze differentiation and mineralization, red positive cells were imaged on an inverted optical microscope (Microscope, Nikon Eclipse using the Leica Applications (LAS EZ, Leica) suites and analyzed using ImageJ. For each condition, 9 spheroids were analyzed. As a control, spheroids of MG-63 (pre-osteoblastic cell line) cells with reduced mineralization and differentiation capacity were analyzed. Osteoblastic-like spheroids were fixed in 4% paraformaldehyde ((PF, Sigma-Aldrich, Vetec) and processed routinely for paraffin inclusion. Paraffin slices were rehydrated and stained with Trichrome Masson (Sigma-Aldrich) and aniline blue (Kit aniline blue, Sigma-Aldrich) that stain collagen fibres^78,79^. For the accomplishment of the histochemical tests, we follow the recommendations of the described kits. Slices were examined and images were captured using an optical microscope (Nikon Eclipse TS100) using the Leica Applications (LAS EZ, Leica). The intensity of the staining of collagen fibres was quantified in histological sections stained with aniline blue using ImageJ (software, 2015) as previously reported. All images were taken and analyzed using exactly the same conditions (exposition time, white balance, background, etc.) with the Nikon software. For each condition corresponding to each experimental group, 9 images of three independent were selected at the area of interest (connective tissue ECM) and the blue staining intensity was automatically calculated by the program.

#### Cytokine assay

After 72 hours and incubation of the spheroids with the TiO_2_ NPs, 100 μL of the supernatant from each condition (control, 5 and 100 μg/mL - 6 samples for each condition) was removed. The supernatant was frozen at −80 °C. Assays were performed following the recommendations of the manufacturer of the MAGPIX reagent kit for identification of cytokines (cytokines -plex panel, Biorad), was employed, capable of quantifying IL-1β, IL-1ra, IL-4, IL-6, IL-7, IL-8, IL-10, IL-12p70, IL-13, IL-15, IL-17, IP-10, IFN-Y, IFN-g, TNFα, VEGF, MIP-1α, MCP-1, PDGF-BB, RANTES, GM-CSF, Eotaxin, Basic FGF, CCL1, CSF3, CSF2, CXCL10, CCL2, CCL3, CCL-4, CCL5. Each step was preceded by washing using the automated washing station (BioPlex-MAGPIX, Biorad). Samples were incubated with detection antibodies.

#### Statistical analysis

Data were presented as mean ± standard deviation (±SD). Gaussian distribution of samples was tested, and the statistical significance of the data was evaluated using One-way ANOVA and unpaired t-test was applied to obtain statistical significance of means. The P value is shown in figures and statistical significance was considered when p < 0.05. Each experiment was performed in three independent experiments with triplicates.

## Supplementary information

Supplementary information
