# Supplementary material for: The two faces of titanium dioxide nanoparticles bio-camouflage in 3D bone spheroids
Source: Sci Rep. 2019 Jun 27;9:9309. doi: 10.1038/s41598-019-45797-6 (PMC6597791; doi:10.1038/s41598-019-45797-6)
Supplement: Supplementary file 1 — Supplementary information [file 41598_2019_45797_MOESM1_ESM.docx]

Supporting Information

**The two faces of titanium dioxide nanoparticles bio-camouflage in 3D bone spheroids**

W. Souza^1,2,3,^ S. G. Piperni^3,4^, P. Laviola^1,3,5^, A.L. Rossi^4^, Maria Isabel D. Rossi^6^, Bráulio S. Archanjo^7^, P.E. Leite^1,2,8^, M. H. Fernandes^9^, L. A. Rocha^3,10^, J.M. Granjeiro^1,2,3,10^A. R. Ribeiro^2,3,5^


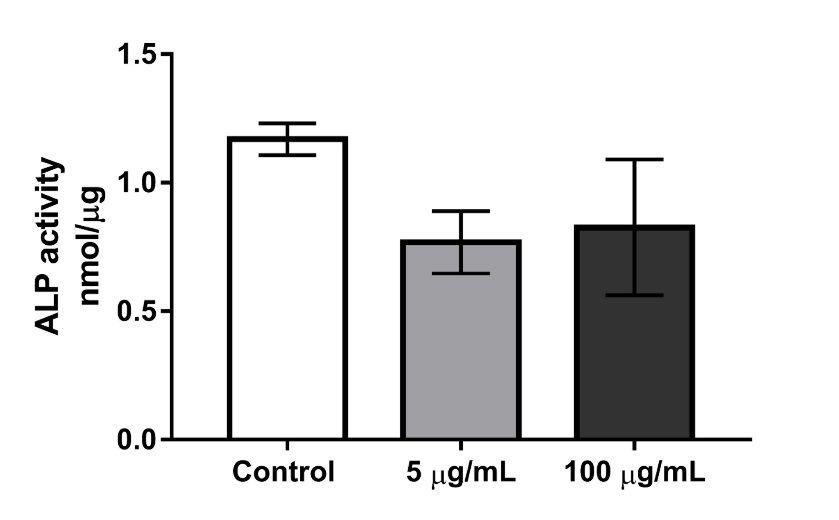


**Complementary Figure 1**: Alkaline phosphatase (ALP) quantification of SAO-2 spheroids upon TiO_2_ NPs exposure comparative to control.


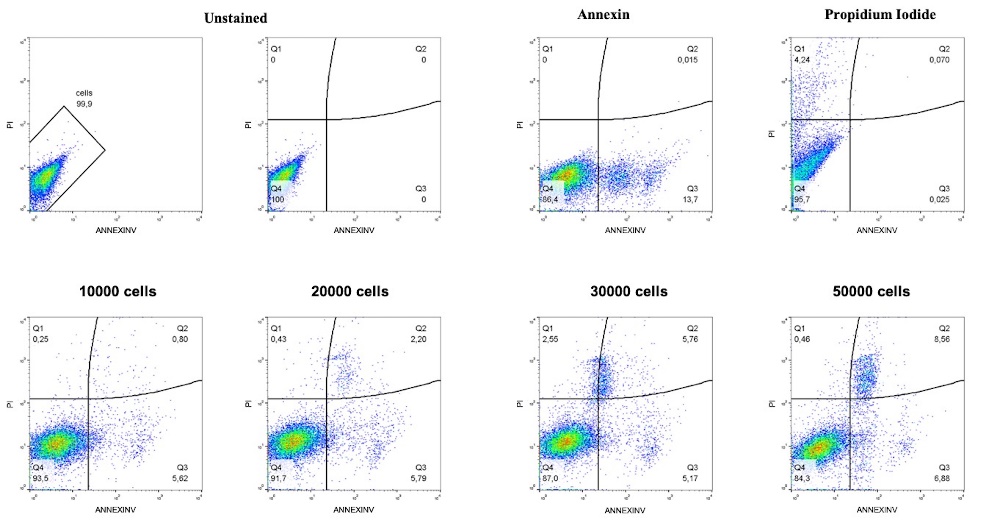


**Complementary Figure 2**: FACS analysis of cell viability in spheroids cultured with different cell numbers with dot plots showing Annexin V and PI expression of cells in spheroids.


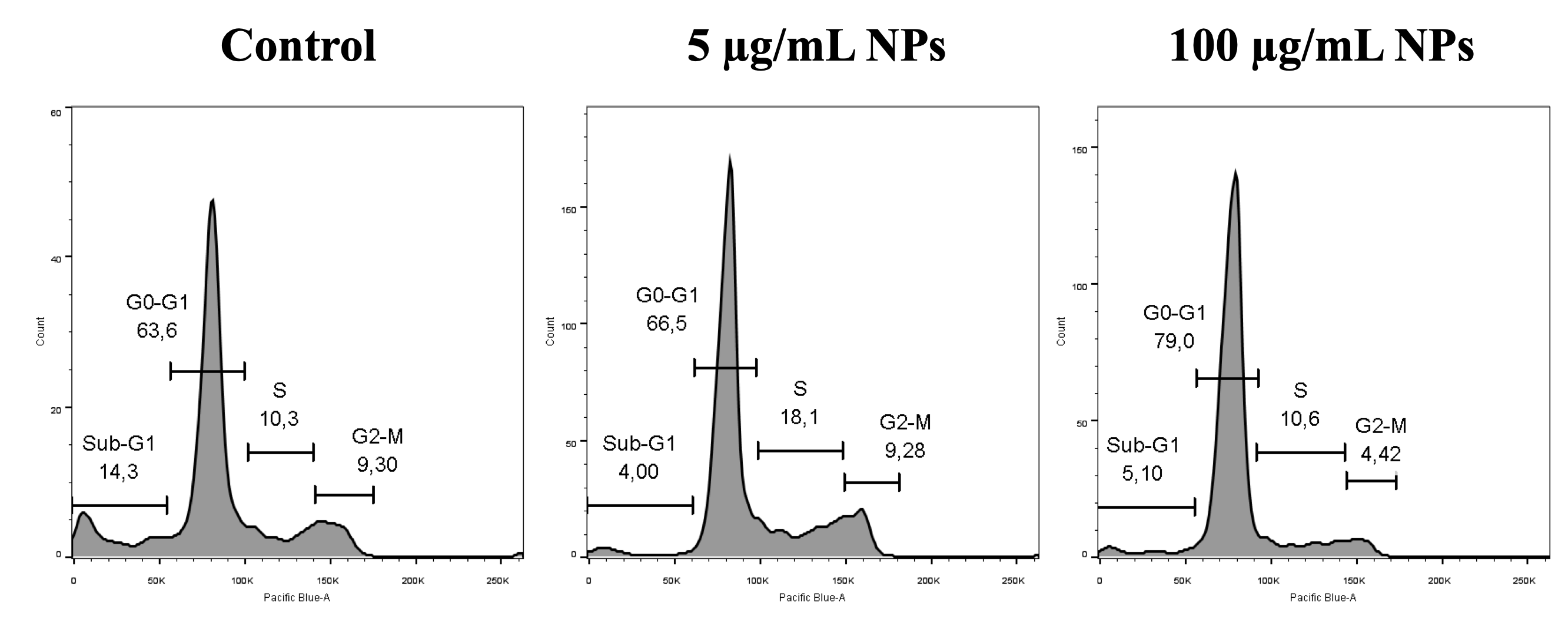


**Complementary Figure 3**: Dot plots showing Annexin V and PI expression of cells in spheroids exposed or not (control) to TiO_2_ NPs and FACS analysis of spheroids cell cycle showing the distribution of each phase.
